# Beyond Traditional Phenolics: Disulfide Bonds for Performance Enhancement of Aerospace Ablation‐Resistant Materials from Processing to Recycling

**DOI:** 10.1002/advs.76868

**Published:** 2026-08-03

**Authors:** Yu Li, Boyuan Hu, Xiaolong Xing, Huan Yang, Ziqi Zhang, Cheng Bian, Ruixue Bai, Ling Yue, Chengshuang Zhang, Xinli Jing

**Affiliations:** ^1^ School of Chemistry Joint Innovation Center of Thermal Protection Materials Xi'an Jiaotong University Xi'an China; ^2^ Xi'an Aerospace Composites Research Institute Xi'an China; ^3^ Xi'an Modern Chemical Research Institute Xi'an China; ^4^ Academy of Aerospace Solid Propulsion Technology Xi'an China

**Keywords:** aerospace ablation‐resistant composites, disulfide bonds, phenolic resin, radicals, renewable

## Abstract

As a key material of solid rocket motor nozzles, phenolic resins (PR) possessing high char yield, good processability, and efficient repairable performance are strongly desired for advanced ablation materials. Achieving these requirements simultaneously is challenging for traditional PR or for dynamically cross‐linked polymers. The present study proposed a strategy to reconcile the thermal stability and reprocessability of ablation materials based on a resole‐type PR bridged with disulfide bonds (HPDS). The aromatic disulfide bonds conferred outstanding performance in resin processing, composite molding, application, and recycling. During processing, the partial dissociation of S─S bonds ensured effective control of the resin's viscosity, allowing for fitting to multiple pre‐impregnated processes. During molding, dissociation and re‐bonding of S─S bonds enabled effective network reconstruction, yielding a repairable resin matrix and allowing high‐value carbon fiber recycling. During the ablation stage, the cleavage of S─S bonds produced long‐lifetime thiyl radicals that captured radicals, protecting carbon‐containing species that participate in char formation. The cured HPDS (CHPDS) exhibited a char yield of 64.7% at 800°C, and its carbon fiber‐reinforced composites are stable to oxyacetylene ablation at 2800°C. From processing to application, from thermal stability to recyclability, the dynamic S─S bonds unleash the renewed vitality of phenolic resins.

## Graphical abstract

The dynamic disulfide bonds facilitated the development of high‐performance aerospace ablation‐resistant materials. At moderate temperature, the dissociation and recombination of disulfide bonds ensured excellent processability, mechanical properties, and recycling ability of phenolic resin; at high temperature, cleavage of disulfide bonds produced thiyl radicals which trapped free radicals during phenolic pyrolysis and significantly improved char yield, enabling good ablation resistance.

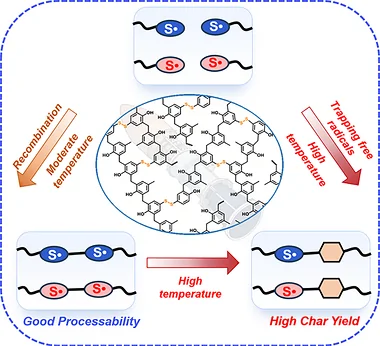

## 1 Introduction

The nozzle serves as the core power component of the Solid Rocket Motors (SMRs), operating under extreme conditions including temperature exceeding 3000°C and high‐speed particle erosion. The ablation resistance of the nozzle expansion section is critical to the reliable performance of the SRM. The expansion section is commonly fabricated with phenolic resin (PR) and carbon fiber (CF) via solution impregnation molding, hot‐melt molding, or resin transfer molding (RTM) [1, 2, 3]. During motor operation, ablation induces pyrolysis of the CF/PR composites, resulting in volatile emissions to release heat and retaining a residual char layer to protect the motor [4]. Given that the CF was fixed, PR with high char yield (usually defined as the weight retention of PR after pyrolysis in an inert atmosphere) is strongly preferred to improve the ablation resistance of the nozzle expansion section [5].

Achieving higher char yield while retaining good processability is the main criterion for PR in advanced ablation composites. Although the theoretical carbon content in PR exceeds 70%, the traditional thermoset PR (Resole) or thermoplastic PR (Novolac) usually exhibits a char yield of ca. 60% or lower. The carbon loss of PR during thermal pyrolysis is mainly caused by cleavage of weak bonds (e.g., ether bonds) and end groups, which produce hydrogen and hydroxyl radicals, further attacking the backbone of PR and leading to carbon loss in the form of carbon dioxide, isolated phenols, or aromatic hydrocarbon compounds [6, 7]. Because the ether bonds and end groups are inevitable in any PR network, the improvement of the char yield usually relies on thermally stable or ceramizable additives. Inorganic compounds containing silicon [8, 9], boron [10, 11, 12], zirconium [13], etc., graphene [14], and carborane [15] have been employed to increase the char yields, while immiscible inorganic compounds generally sacrifice processability of PR for composite preparation. Recently, more efforts were put on modifying PR with organic compounds or designing new synthetic routes, in order to reconcile the char yield and mechanical properties of a composite. We synthesized a resole‐type PR with bisphenol F and formaldehyde, where the integrity of the crosslinking network was manipulated to diminish ether bonds, and PR with char yield, exceeding 66% was achieved without introducing external additives [16]. By precisely regulating the pH at a critical reaction stage of phenol‐formaldehyde polymerization, Yang et al. acquired a low‐viscosity PR with excellent processability. After a segmental curing process, this kind of PR displayed a char yield as high as 72% [17]. Introduction of carbon‐rich groups such as naphthalene [18] or fluorene [19] was also found to improve char yield [19]. Nevertheless, none of these approaches address the issue of repairability or recyclability, and are incapable of preparing reparable composites.

The key challenge is that repairing the composite or recycling the resin requires a reversible or reconfigurable crosslinking network, whereas a high char yield requires an integrated and stable network. The manufacture of the expansion section of rocket nozzle relies on time‐consuming and high‐energy‐consumption procedures. Due to fluctuation of volatiles content in the prepregs, winding status, and resin fluidity during curing, once defects occur in the expansion section, the product is usually discarded and cannot be reused, leading to huge economic loss. The utilization of biomass resources [20, 21] and dynamic covalent bonds [22, 23, 24, 25] indeed gives rise to a lot of renewable or healable PRs, whereas most of these PRs displayed poor char yield and were unable to be used as ablation‐resistant materials. The Novolac‐type PR crosslinked with boric ester bonds can be completely recycled in ethanol, while the high content of reversible boric ester bonds requires a duration as long as 30 h for curing [26]. By synergistically crosslinking Novolac resin with both phenol boric acid and hexamethylenetetramine (HMTA), the network based on dynamic boric ester bonds and methylene groups ensured high char yield, moderate curing temperature, and good reparability [27]. However, the resin containing a certain amount of the boric ester bonds was too sensitive to moisture, bearing poor storage stability. Recently, Zhang et al. reported a reprocessable silicon‐containing phenolic resin via the hybrid exchange reaction between Ph─OH and Si─O─Si bonds. The exchange reaction enabled full curing of the resin, which displayed a high thermal degradation temperature and char yield of 63.7% at 800°C [22]. Nevertheless, in contrast to plenty of reports on repairable and reprocessable epoxy with improved thermal resistance [28, 29], achieving repairable and renewable PRs for ablation‐resistant composites is relatively rare [30, 31]. This is the gap we aim to fill.

With the understanding of the PR pyrolysis mechanism and the exchange mechanism of dynamic covalent bonds, the disulfide bonds—a commonly studied reversible bond with low bond energy—caught our attention. On the one hand, the dynamic dissociation/exchange of disulfide bonds can take place far below the thermal degradation temperature of PR (e.g., 300°C), which will enable post‐cure repair and recycling of PR. On the other hand, during thermal degradation, the cleavage of S─S bond, which may take place before C─C bond and generates long lifetime thiyl radicals. These radicals are supposed to capture other radicals and escape in the form of volatiles, thereby suppressing attack on the resin backbone and reducing carbon loss. The disulfide bonds have been widely employed to synthesize repairable thermosets (e.g., epoxy resins [32, 33, 34]), elastomers [35, 36, 37], hydrogels [38], or adhesives [39] by utilizing their reversible dissociation/exchange behaviour, whereas the roles of sulfur radicals on ablation materials was rare concerned.

Here, we report a sulfur‐containing resole‐type PR (noted as HPDS) synthesized with a methylol compound, 2,6‐Bis(hydroxymethyl)‐*p*‐cresol (HPC), and 4,4'‐disulfanediyldiphenol (DPDS). The HPDS resin simultaneously achieves: (a) good processability, (b) high char yield, (c)efficient reparability, and (d) considerable ablation resistance — properties that are typically mutually exclusive in conventional PRs or other dynamic‐bond‐based PRs.

## 2 Results and Discussion

### 2.1 Composition and Reactivity of HPDS

The HPDS was a reactive mixture containing HPC‐substituted DPDS derivatives, oligomers of HPC, and the residual HPC and DPDS. At the synthetic temperatures (100∼110°C), DPDS behaved similarly to a normal biphenol (e.g., 4,4'‐Dihydroxydiphenylmethane, BPM) and underwent condensation reactions with hydroxymethyl groups in HPC, resulting in a liquid active mixture (Figure 1a). Take HPDS‐1.7 as an example; the ^1^H NMR spectrum of the reaction products showed that the H atom signal for the methylene group (position **a**, 4.02 ppm) between the phenolic rings increased with an extension of reaction time (Figure 1b). Simultaneously, the signal attributed to the methylene protons in the hydroxymethyl groups (position **b**, 4.72 ppm) diminished, confirming the occurrence of condensation. The condensation between the hydroxymethyl groups (─CH_2_OH) of HPC and the *ortho* hydrogen in the phenol ring of DPDS results in a series of HPC‐substituted DPDS derivatives (Figure 1c). The average molecular weight of the products gradually increased with elongation of reaction time (Figure 1d). After 0.5 h of reaction duration, a clear pale‐yellow liquid was obtained with a molecular weight of *ca*. 544 g·mol^−1^ and viscosity at the 10^2^ Pa·s level, which can be directly used as a kind of thermoset PR (noted as HPDS‐1.7, Table S3 and Equation S1).

**FIGURE 1:**
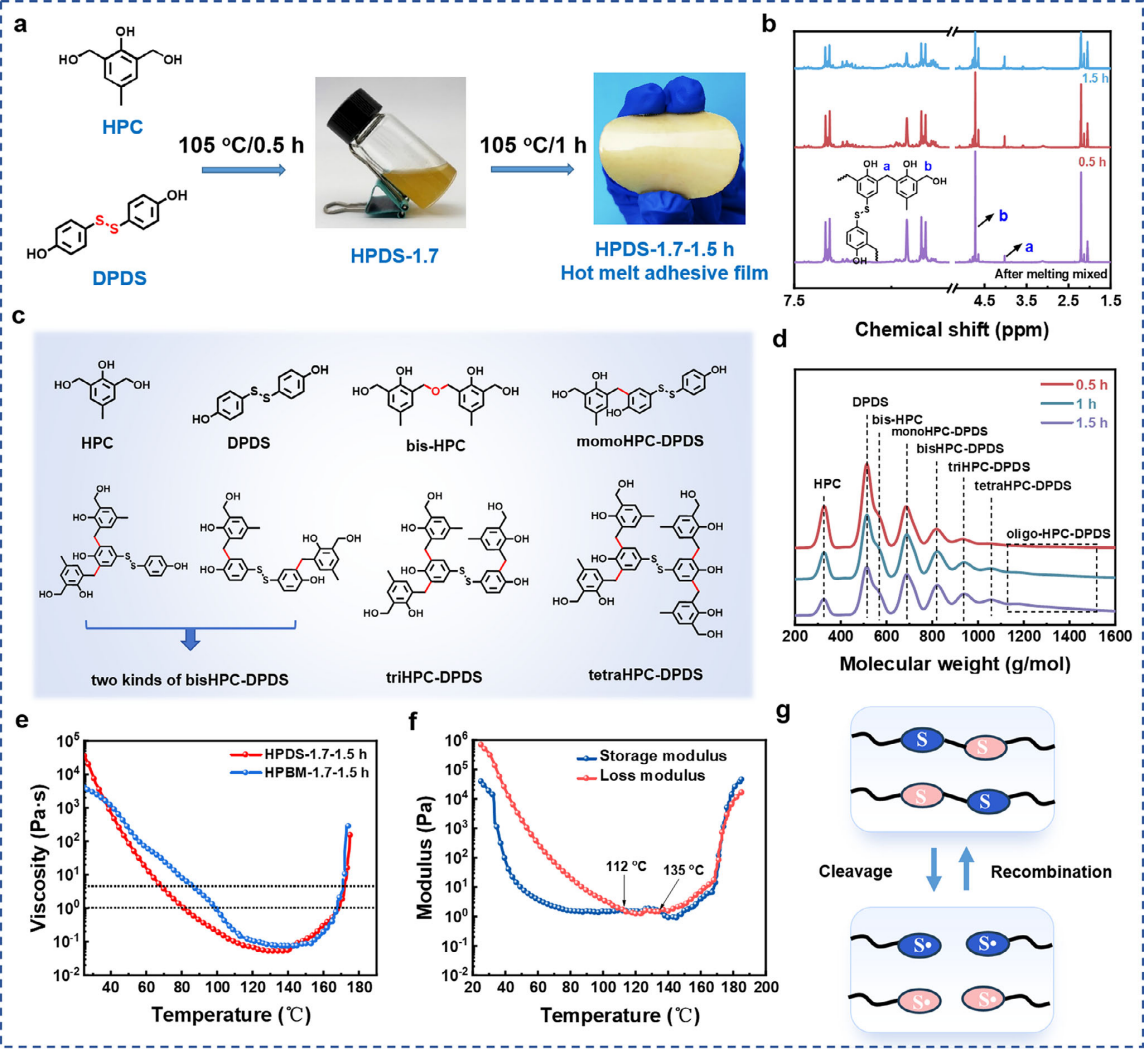
Synthesis, structure, processability and curing of HPDS and HPBM. (a) Schematic illustration of synthesis of HPDS with HPC and DPDS; (b) ^1^H NMR (Acetone‐d_6_) spectra of the mixture of HPC and DPDS after reaction duration for 0.5 h and 1.5 h at 105°C; (c) Possible main species in HPDS; (d) GPC curves of HPC and DPDS reaction products after reaction duration for 0.5 h, 1 h and 1.5 h at 105°C; (e) Demonstration of the processability of HPDS‐1.7‐1.5 h and HPBM‐1.7‐1.5 h with viscosity‐temperature relationship (heating rate: 2°C∙min^−1^); (f) The modulus change of HPDS‐1.7–1.5 h upon heating (frequency: 1 Hz; heating rate: 2°C∙min^−1^); (g) Schematic illustration of reversible transformation between disulfide bonds and thiyl radicals.

The lower reactivity of DPDS (as compared with phenol or BPM) and the dynamic dissociation behaviour of thiyl bonds resulted in a slower increase in viscosity during the polycondensation reaction, rendering it suitable to regulate the processing ability. Due to the electron‐withdrawing effect of the S atom on phenol rings, the electron density on the *ortho*‐carbon relative to hydroxyl groups in DPDS was slightly lower than that in BPM (Figure S3), attributable to the electron‐withdrawing effects of S atoms. HPDS synthesized with a reaction duration of 0.5 h exhibited a viscosity of ca. 287 Pa·s at 30°C, and this viscosity decreased to less than 1 Pa·s with an increase in temperature to 80°C and was maintained lower than 1 Pa·s until 160°C (Figure S5a). Even at 120°C, HPDS‐1.7 maintained a viscosity of below 1 Pa·s over 2 h (Figure S5b). With an increase in temperature, the condensation reaction proceeded faster and led to an increase in viscosity; however, the dissociation of S─S bonds was also promoted, leading to a slow increase in viscosity. This feature renders HPDS‐1.7 unique from traditional resole‐type PR and is well‐suited for RTM technology, which typically requires the resin to exhibit low viscosity (<1 Pa·s) during injection and possess an adequate pot life [40].

As the reaction duration was controlled for 1.5 h, the product (noted as HPDS‐1.7–1.5 h) exhibited high viscosity up to 10 ^4^ Pa·s at room temperature, while its viscosity decreased by 3–4 orders of magnitude at elevated temperatures (Figure 1e). The average molecular weight of HPDS‐1.7‐1.5 h increased to ca. 688 g·mol^−1^ with the residual free HPC obviously reduced (Figure 1d; Table S3). The moderate molecular weight and viscosity render the material a flexible film at room temperature (Figure 1a). With an increase in temperature to 65–70°C, the viscosity of HPDS‐1.7–1.5 h decreased to 5–20 Pa·s, and the low viscosity was maintained for greater than 80 min (Figure S6). During the curing process, HPDS‐1.7 gradually forms a three‐dimensional cross‐linked network, which hinders the movement of molecular chains. When the temperature increased to around 120°C, HPDS‐1.7 displayed its storage modulus exceeding the loss modulus (Figure 1f). Notably, the loss modulus started to be lower than the storage modulus at approximately 140°C, which can be attributed to reversible dissociation/exchange of disulfide bonds (Figure 1g). This feature renders HPDS‐1.7‐1.5 h well‐suited to the requirements of the hot melting processing method of PR‐based composites [41]. For comparison, by replacing DPDS with BPM, the corresponding product, HPBM‐1.7‐1.5 h, exhibited a viscosity lower than 10^4^ Pa·s at room temperature (Figure 1e), while its gelation time (the time required for the resin to transform from a flowing liquid state into a solid gel at a specified temperature) measured at a curing temperature of 165°C was much shorter than that of HPDS‐1.7–1.5 h, making it less suitable for hot‐melt technology.

### 2.2 Crosslinking of HPDS

Despite the lower reactivity of the *ortho*‐position of DPDS toward hydroxymethyl compared to normal phenol rings, a higher crosslinking degree was achieved for HPDS than for its counterpart HPBM under the same curing condition at 165°C for 2 h, owing to the dynamic nature of S─S bonds (Figure 2a). Based on curing kinetics analysis, the curing temperature range of HPDS‐1.7 was determined to be from ca. 131.5°C to 212°C (Figure S7a,b), and the curing peak temperature for HPDS‐1.7 was determined around 177°C, higher than that of HPBM‐1.7 (173°C, Figure S8a,b). Accordingly, the average apparent activation energy for the HPDS‐1.7 curing reaction (110.2 kJ·mol^−1^) was slightly higher than that of HPBM‐1.7 (99. 4 kJ·mol^−1^) [42] (Tables S4 and S5). At the fixed curing temperature (e.g., 165°C), the gelation times for HPDS‐1.7‐1.5 h and HPBM‐1.7‐1.5 h were 25.13 min and 17.63 min, respectively. After curing at 165°C for 2 h, CHPDS‐1.7 reached a curing degree of 98.7% (Figure S9 and Table S6), considerably higher than its counterpart CHPBM‐1.7 (76.4%; Figure S10), indicating that CHPDS‐1.7 achieved nearly complete curing, probably resulting from disulfide bond dissociation enhancing polymer chain mobility. CHPDS‐1.7 exhibited a gel fraction up to 99% (Figures S11 and S12) based on solvent swelling, and its DSC curves revealed a glass transition temperature (T_g_) at 140.6°C without other detectable heat effects, further indicating completion of the curing reaction (Figure 2b). The DMA results showed that CHPDS‐1.7 maintained a storage modulus of approximately 2.5 GPa within the range of 30–120°C and decreased to 10^1^ GPa after glass transition, confirming its rigid but highly cross‐linked structure (Figure 2c).

**FIGURE 2:**
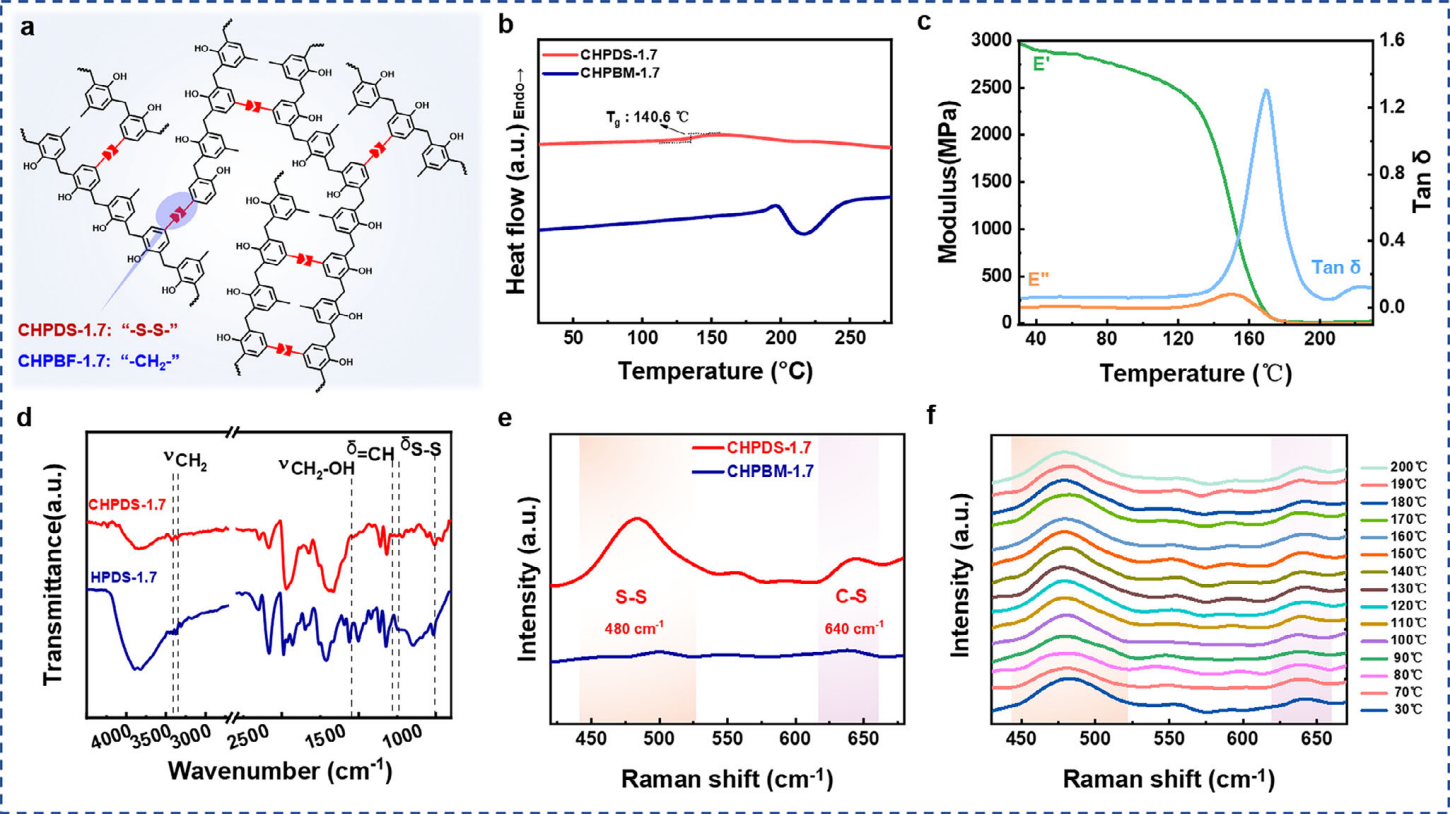
(a) Schematic illustration of the network structures of CHPDS‐1.7 and CHPBM‐1.7; (b) DSC curves of CHPDS‐1.7 and CHPBM‐1.7; (c) DMA testing of HPDS‐1.7 was performed under a constant heating rate (3°C∙min^−1^); (d, e) FTIR and Raman spectra of HPDS‐1.7 before and after curing (noted as CHPDS‐1.7); (f) The temperature‐dependent Raman spectra of CHPDS‐1.7 (heating rate: 3°C∙min^−1^; holding time: 5 min).

The CHPDS‐1.7 formed a crosslinking network containing phenol rings bridged with disulfide bonds and methylene groups. The disappearance of the absorption peaks associated with the C─O stretching vibration in the FTIR spectrum (1064 cm^−1^) indicates consumption of the hydroxymethyl groups in HPC (Figure 2d; Figure S2). Both the FTIR spectrum (presence of S─S vibration at 516 cm^−^^1^ [43]) and Raman spectrum (480 cm^−^^1^ for S─S and 640 cm^−^^1^ for C─S [44]) verified successful retention of S─S bonds in the cured sample, indicating the stability of S─S bonds even after 2 h heating treatment at 165°C (Figure 2e). Upon heating, a clear signal belonging to S─S bonds was still detected up to 200°C (Figure 2f). Notably, in the DMA curves, after the glass transition, a shoulder peak appeared in the tan δ value of HPDS‐1.7 around 220°C, attributable to the dissociation of S─S bonds (Figure 2c).

### 2.3 Thermal properties of CHPDS

During the pyrolysis of PR, the cleavage of disulfide bonds produced sulfur radicals, which suppressed weight loss at higher temperatures, resulting in significantly improved char yields. The thermal stability of CHPDS‐1.7 was evaluated by TGA in comparison with its counterpart, CHPBM‐1.7. In the first temperature stage (from room temperature to 250°C), almost no weight loss was detected for either sample, indicating their integrated crosslinking structure (Figure 3a). In the second stage (from 250°C to 400°C), the cleavage of covalent bonds, e.g., C─C, C─H, C─O, and S─S bonds, occurred, producing volatiles including water, carbon dioxide and isolated phenols, as indicated by the drastic weight loss in the TGA curves. It was known that the C─C bonds on the main chain of phenolic resin underwent even cleavage above 300°C while the breakage of the C─C bonds at the chain ends was the main cause for drastic weight loss [6]. Due to the weaker bond energy of the S─S bond (Figure S16), the S─S bond at the chain end breaks before the C─C bond, resulting in the thermal decomposition temperature (e.g., T_d5%_) of CHPDS‐1.7 being lower than that of CHPBM‐1.7 (Figure 3a). However, in the third stage (from 400°C to 800°C), the weight loss of CHPDS‐1.7 was obviously impeded, as shown in the DTG curve (Figure 3b), and the weight retention of CHPDS‐1.7 was higher than that of CHPBM‐1.7, resulting in a char yield of 64.7% for CHPDS‐1.7, significantly higher than that of CHPBM‐1.7 (54.4%). Considering the lower crosslinking density of CHPBM‐1.7, it was further cured at 195°C for another 2 h after being cured at 165°C for 2 h, and its crosslinking density increased to 94.6% (Figure S10 and Table S6). Accordingly, the char yield of the post‐cured CHPBM‐1.7 increased to 57.3%, whereas it is still significantly lower than that of CHPDS‑1.7 (Figures S14 and S15). This indicates that reversible disulfide bonds can promote the formation of a complete crosslinking network in phenolic resins, which is beneficial for enhancing the char yield. As the dosage of DPDS was slightly decreased in order to avoid excess weight loss during the second stage (with sulfur content decreasing from 11.72% to 10.71%), the char yield of CHPDS‐2.0 was further increased to 65.9% (Table S7). Notably, the char yield of CHPDS‐2.0 exceeded the reported PRs for ablation applications [45, 46, 47], including some of the silicon, zirconium modified PRs [48, 49, 50], while CHPDS‐1.7 resin combines high carbon yield and processability.

**FIGURE 3:**
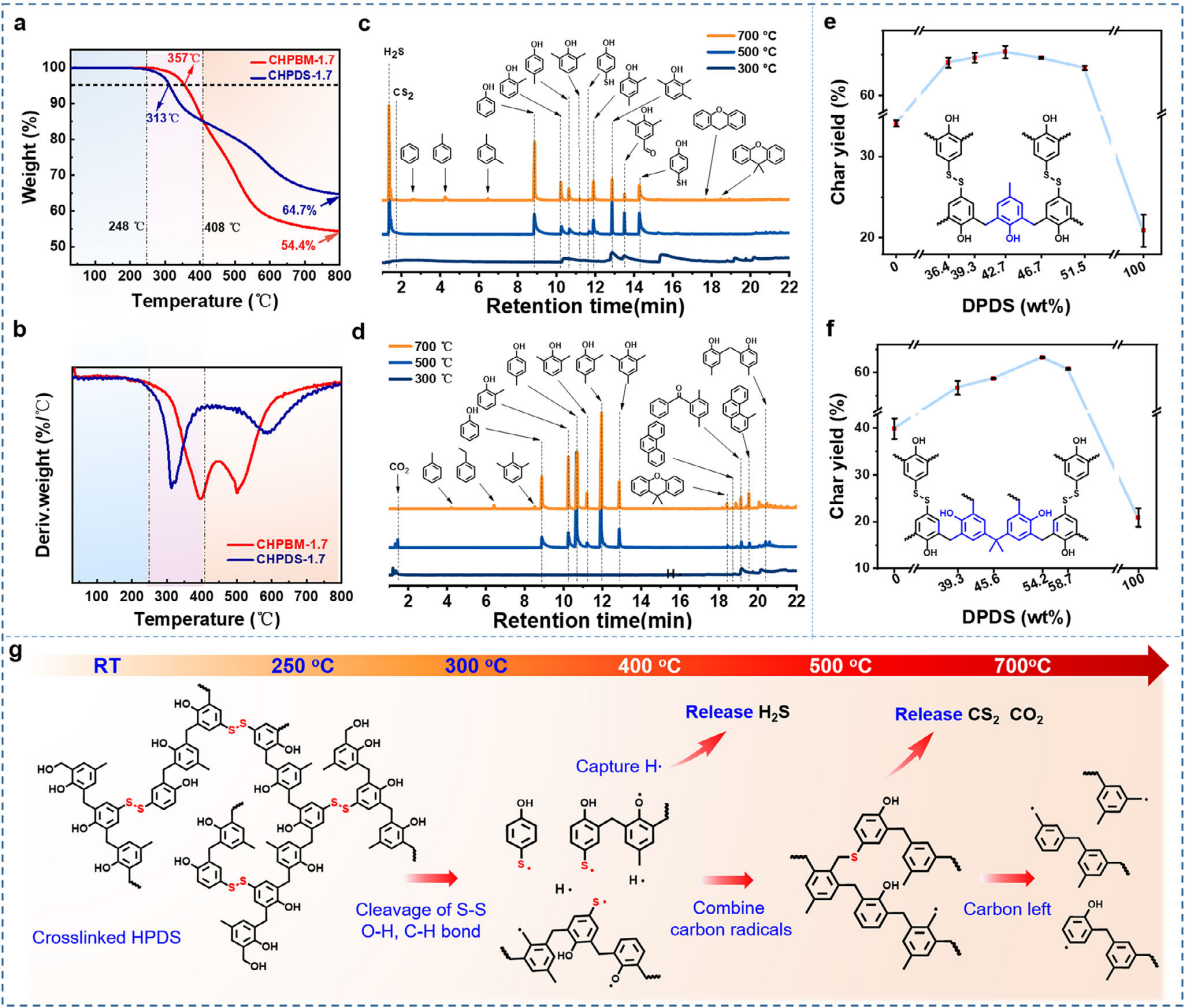
Thermal analysis of CHPDS‐1.7 and CHPBM‐1.7. (a, b)TG and DTG curves of CHPDS‐1.7 and CHPBM‐1.7; (c, d) The possible pyrolysis products of CHPDS‐1.7 (or CHPBM‐1.7) collected at 300°C, 500°C and 700°C, as analyzed with Py‐GC/MS; (e, f) Char yield of CHPDS and CBHDS with different DPDS contents at 800°C; (g) Schematic illustration of sulfur‐containing compounds evolution during the pyrolysis of CHPDS.

The higher char yield of CHPDS can be attributed to the thiyl radicals formed during the early stage of CHPDS pyrolysis, which help retain carbon elements through combination with carbon‐containing radicals, whereas the sulfur element itself mainly escaped as H_2_S and CS_2_ at elevated temperatures (Figure 3c,g). The low energy of aromatic disulfide bonds (Figure S16) enabled thiyl radical formation even under mild conditions. For HPDS‐1.7, after curing at 165°C for 2 h, its Electron paramagnetic resonance (EPR) spectroscopy clearly displayed a signal corresponding to the spin of free radicals, whereas in raw DPDS and CHPBM‐1.7, almost no signals could be detected (Figure S17). Herein, the CHPDS network with high T_g_ is beneficial to trap these thiyl radicals and protect them from pairing up to form disulfide bonds again [51]. An increase in the thermal treatment duration induced a decline in the free radical spin signal because of thermal quenching, whereas even after curing at 165°C for 2 h followed by treatment at 180°C for 30 min, remarkable spin signals can still be detected in CHPDS, indicating the extended lifetime of sulfur radicals (Figure S17). During thermal degradation, the cleavage of S─S bonds occurs prior to C─C bond cleavage, leading to a lowered decomposition temperature for CHPDS‐1.7 compared with that of CHPBM‐1.7 (Figure 3a).

Nevertheless, the formation of thiyl radicals led the following situation in two directions, on the one hand, a part of the produced thiyl radicals (S•) combine with hydrogen radicals (H•), forming H_2_S gas that escapes from the system, as indicated by Py‐GC/MS results (Figure 3c,g); thus, the H• that attacks the backbone of PR was removed in some extent. On the other hand, the S• can still capture the thermally generated C• to form more stable S─C bonds (71.36 kcal·mol^−1^), leaving more opportunities for carbon atoms to participate in the char formation. In the pyrolysis products of CHPDS, the amount of phenols and polycyclic aromatic hydrocarbons was drastically reduced compared with CHPBM (Figure 3c,d). It is further deduced that once more carbon radicals are present, a higher content of disulfide bonds will be required to retain carbon. In a parallel study, HPC was replaced with 2,2‐Bis[4‐hydroxy‐3,5‐bis(hydroxymethyl)phenyl]propane (BHP), which can be considered an analog of a HPC dimer, to react with DPDS, and the product was denoted as BHDS (Scheme S2). In BHP, larger carbon‐containing radicals will form in comparison with HPC; consequently, the highest char yield of BHDS (63.2%) was achieved when the dosage of DPDS was 54.2%, corresponding to a sulfur content of 13.62% (Figure 3f; Table S9). Similarly, for the HPDS*‐x* with *x* greater than 2 (less DPDS used), the incorporated disulfide bonds were insufficient to generate sulfur radicals, leading to deceasing of the char yield of CHPDS*‐x* (Table S7). In other words, thanks to the presence of S─S bonds, the weight loss due to escape of carbon‐containing species from collapse of the crosslinked PR network was retarded. The elemental analysis also revealed that CHPDS‐1.7 exhibited a higher carbon retention ratio even after heat treatment (800°C for 2 h) compared with CHPBM‐1.7, indicating its superior ability to fix carbon elements within the char structure during the carbonization process (Table S10).

In order to further verify the contribution of disulfide bonds, DPDS was introduced to a commercially available barium‐catalyzed phenol‐formaldehyde resin (noted as PF), and cured under the same conditions as those for HPDS (noted as CPFDS). It was found that, with the sulfur content of 9.5%, the CPFDS‐1.7 displayed a char yield of 65.7%, which is significantly higher than that of the pristine cured PF (63.7%). Accordingly, with a higher content of sulfur involved, the excess unstable disulfide bonds eventually decreased the char yield (Table S11 and Figure S20), reflecting the specific roles of thiyl radicals in phenolic resin.

### 2.4 Dynamic Behavior of HPDS Cross‐Linking Network

Benefiting from the dynamic nature of disulfide bonds, the CHPDS network possessed both an integrated structure and the remarkable reconstruction ability, enabling good mechanical strength, effective reprocessing, and healing ability for the resin block and its fiber‐reinforced composites. Notably, traditional PR was typically too brittle to measure the tensile strength of the resin block, and few of the pure resin's strength has been reported. The CHPDS‐1.7 resin with dynamic disulfide bonds makes it easy to be molded as a pure resin block, which exhibited excellent mechanical properties, with a tensile strength as high as 42.3 MPa, a tensile modulus of 2.0 GPa, and an elongation at break of 3.5% (Figure 4a; Figure S17a and Table S12). The tensile strength of CHPDS‐1.7 was extraordinarily higher than other dynamically crosslinked PRs [22, 23, 25]. With decreased DPDS feeding ratio, the tensile strength of CHPDS‐2.0 further reached 61.7 MPa, attributable to its higher crosslinking density (Figure S21b and Table S12). In the temperature range of approximately 185°C to 200°C, CHPDS‐1.7 displayed significant stress relaxation, with a characteristic relaxation time (τ) of less than 15 min (Figure 4b), and the corresponding relaxation activation energy was calculated as 142.9 kJ·mol^−1^ (Equation S7). Though this relaxation activation energy was higher than that of other vitrimers [52, 53] containing S─S bonds due to the rigid and aromatic backbone of PR, the rapid stress relaxation reflected the strong rearrangement ability of the CHPDS network, confirming its enhanced processability. The cracks on the surface of the CHPDS‐1.7 block could be completely repaired after pressing at 210°C for 1 h (Figure 4c). After use, the broken CHPDS‐1.7 can also be renewed through mechanical recycling, e.g., by grinding the resin pieces into fine powder and hot pressing at 210°C (*ca*. 8 MPa). The reprocessed CHPDS‐1.7 was still transparent and retained 77.5% of its original tensile strength, indicating its homogeneous state (Figure 4a).

**FIGURE 4:**
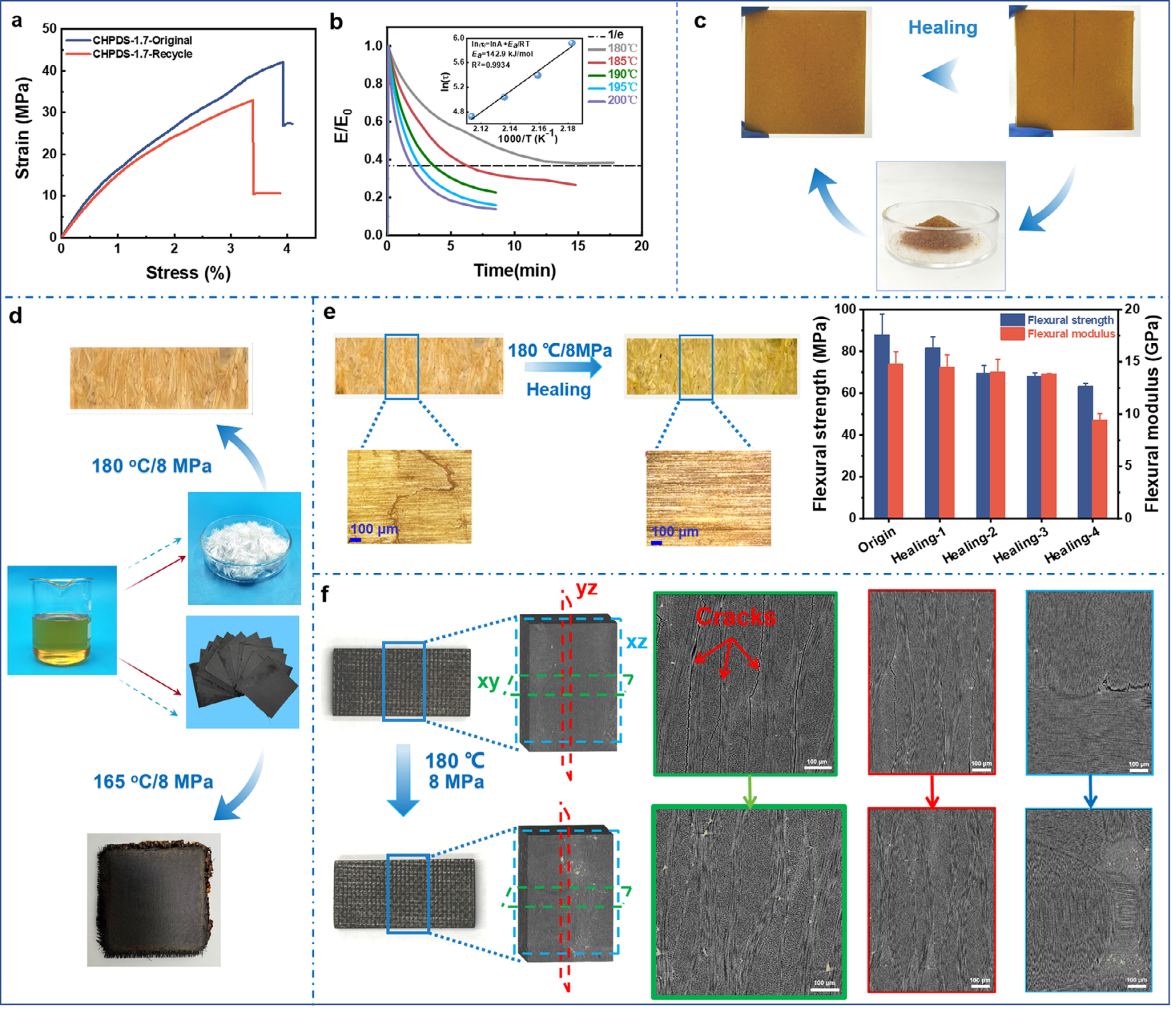
The network reconstruction ability manifested in the CHPDS resin block and the composites. (a) The stress‐strain curves of CHPDS‐1.7 before and after reprocessing; (b) Stress‐relaxation behavior of CHPDS‐1.7 at different temperatures; (c) Snapshots for repairing and remolding of CHPDS‐1.7 block; (d) Preparation procedure of CGF/HPDS‐1.7 and CF/HPDS‐1.7; (e) Healing of the CGF/HPDS‐1.7 composite and its mechanical properties CGF/HPDS‐1.7 after each repairing cycle; (f) Micro‐CT images for detection of CF/HPDS‐1.7 with interlaminar damage before and after hot‐pressing repair.

The good reconstruction ability ensured high healing efficiency for both the short and long fiber‐reinforced HPDS composites. During manufacturing, the formation of defects or cracks in the composite materials is inevitable, whereas the dynamically crosslinked network of CHPDS‐1.7 enabled the repair of composites even after curing (Figure 4d). For instance, when a CGF/HPDS‐1.7 molding composite was damaged during a bending test, the crack could be effectively healed by directly hot pressing the specimen (Figure 4e). The healed specimen retained 92.7% of its original flexural strength (Figure S22). Even after four healing cycles, the specimen maintained 72.0% of its original flexural strength, and the bending modulus remained basically unchanged after the first three healing cycles (Figure 4e). Notably, the repaired sample became slightly yellow‐green, a result of disulfide bond oxidation during the hot‐pressing process. The decreased healing efficiency after multiple breaking‐molding cycles is attributable to partial fiber breakage and the loss of some disulfide bonds.

The CF/PR composites are the main component in current solid rocket expansion sections, requiring both high ILSS and low ablation rates. Benefiting from well‐constructed curing structures, the CF/HPDS‐1.7 composite exhibited superior mechanical properties compared with other carbon fiber‐reinforced PR matrix composites, with an ILSS of 53.2 ± 1.2 MPa (Figure S23). Furthermore, the CF/HPDS‐1.7 samples with interlaminar damage could be repaired via hot pressing. As shown in Figure 4f, tiny interlaminar cracks were pre‐made in a CF/HPDS‐1.7 specimen through slight bending, and all the cracks were subsequently healed after hot pressing the specimen. The Micro‐Computed Tomography (micro‐CT) images showed that, after healing, defects in the *xy*, *yz*, and *xz* directions disappeared, leaving compact cross sections (Figure 4f). Due to different degrees of fiber breakage in samples with interlaminar damage, the repair efficiency of CF/HPDS‐1.7 varies significantly (Table S13). Considering the complicated and expensive manufacturing of the expansion section, the healing ability of CHPDS‐1.7 provides a solution to reduce defects and improve the production efficiency of the composites.

### 2.5 Chemical Recycling of CHPDS‐1.7 and Recovery of Carbon Fibers

With disulfide bonds incorporated, the CHPDS‐1.7 composites degraded into soluble thermoplastic PR in strong polar solvents under thermal stimuli (Figure 5a), which is useful to recycle high‐value carbon fibers from the composites. Currently, once defects appeared in the expansion sections made of normal PR and CF, the products are typically discarded, leading to considerable economic costs. Alternatively, after swelling the CF/HPDS‐1.7 composites in N, N‐Dimethylformamide (DMF) and heating at 140°C for 12 h, the resins were thoroughly exfoliated from the CF in the bulk composite specimen (Figure 5b). The recovered CF cloth showed a smooth and clear surface, close to pristine CF cloth. During this process, the strong polar solvent and heat stimuli could accelerate the dissociation and exchange of S─S bonds [54], thus, the cross‐linked network collapsed into soluble fragments (Figure 5a). Structural analysis of the degraded products showed that S─S bond almost disappeared, whereas sulfur mainly formed S─H bond, indicated by the emergence of a thiol stretching vibration peak at 2660 cm^−^^1^ and the XPS results (Figures S24–S26) [55].

**FIGURE 5:**
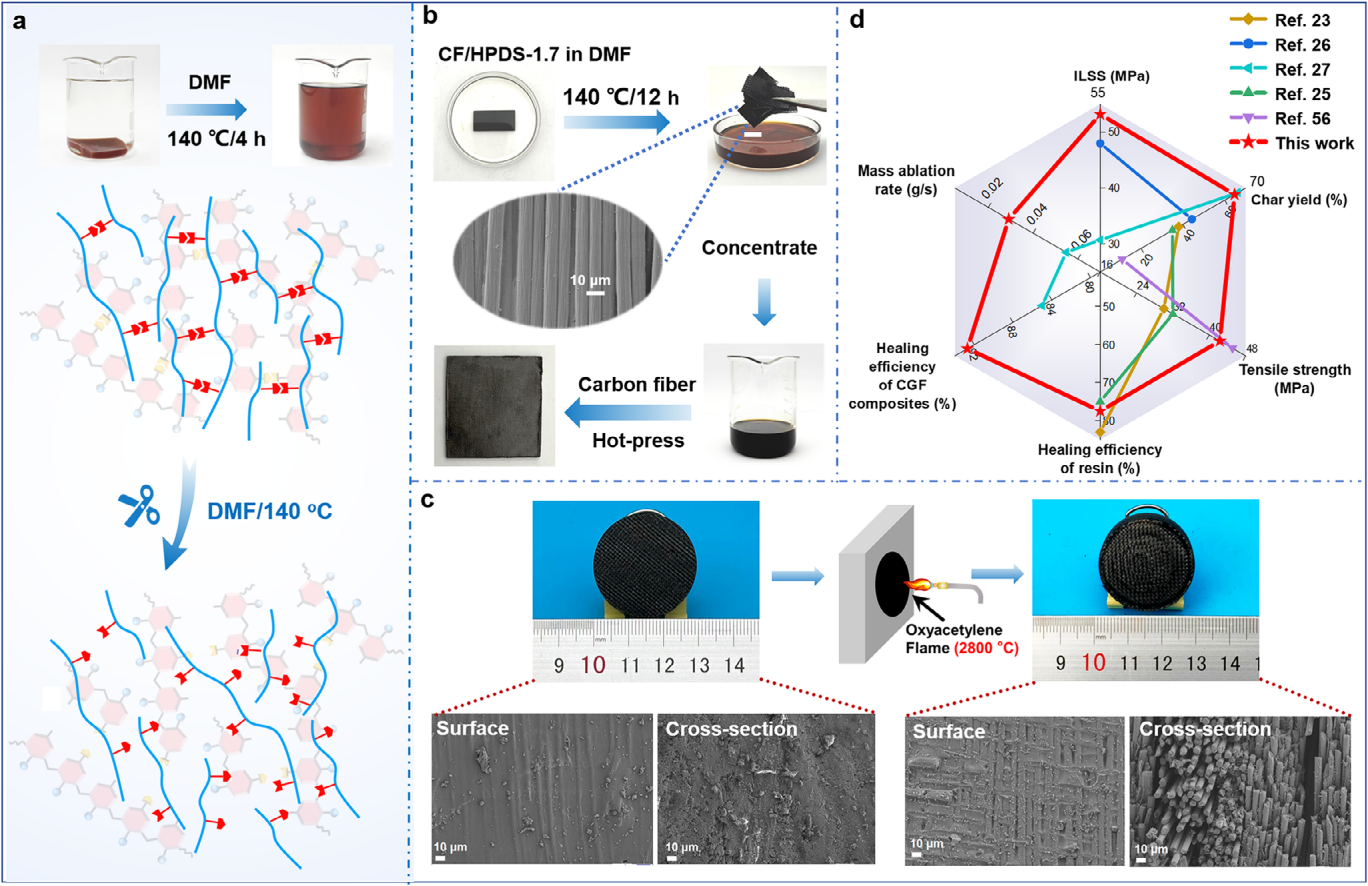
(a) Schematic illustration of CHPDS‐1.7 network change during chemical recycling; (b) Recovery of carbon fiber cloth from CF/HPDS‐1.7 after 12 h degradation; (c) Ablation of CF/HPDS‐1.7 upon oxy‐acetylene flame at 2800°C and the surface morphology before and after ablation; (d) Comparison of the mechanical and thermal properties of CHPDS‐1.7 and its composites with typical literature result [23, 25, 26, 27, 56].

The degraded CHPDS‐1.7 can be re‐cured to produce renewable composites. For instance, by removing excess DMF through rotary evaporation, the concentrated degradation solution can be directly brushed onto CF cloth to prepare the prepregs (Figure 5b). After hot pressing, the recycled CF/CHPDS‐1.7 composite was obtained, which exhibited an ILSS of 15.8±2.4 MPa. The loss of mechanical strength is reasonable, since the cleavage of S─S bonds restricted the reconstruction of a complete crosslinking network. Nevertheless, the degradation products can be further crosslinked by introducing additional hydroxymethyl groups, e.g., using a traditional curing agent, HMTA, to be used as a matrix for molding compounds (Figure S28).

### 2.6 Ablation Resistance of Carbon Fiber‐Reinforced Composite Materials

With a high char yield, integrated crosslinking structure and good mechanical strength, the CF/HPDS‐1.7 composite exhibited excellent stability during oxy‐acetylene ablation at 2800°C. After 20 s of ablation, all the composites showed a compact and integrated surface, free of any notable collapse or voids (Figure S29); thus, exhibiting significant potential for ablation protection applications. Ablation resistance was evaluated by calculating the mass or size reduction per second. The mass ablation rate was measured as 0.029 g·s^−1^ (Table S14), considerably lower than that of boron‐containing PR (0.055 g·s^−1^) or resole‐type PR with an optimized cross‐linking structure (0.046 g·s^−1^) [16]. The large reduction in mass ablation rate results from disulfide bond‐induced increases in the char yield, which effectively prevent the ingress of oxygen, combustible gases, and heat. Moreover, the escape of sulfur‐containing volatiles can also remove heat and weaken the ablation degradation. As monitored during long‐time ablation with butane, the backside temperature of CF/HPDS increased more slowly than that of CF/traditional PR composite (Figures S30 and S31). With a decrease in the sulfur content, corresponding to a higher HPC content, the mass ablation of CF/HPDS‐2.3 increased (0.039 g·s^−1^), probably resulting from ether bonds due to excessive hydroxymethyl groups. Indeed, for CF/HPDS‐1.7, the higher content of dynamic disulfide bonds may induce expansion of the composite during ablation; thus, the linear ablation rate was measured as negative values. The negative linear ablation rate may be caused by volatile escaping, a small ablation heat flux or weak interlaminar strength [8]. Herein, this can be mainly attributed to the dynamic nature of disulfide bonds in CHPDS‐1.7, which endows the resin with certain plasticity. As a result, the material lacks sufficient mechanical strength to resist the internal stress and the deformation caused by evolving pyrolysis gases during ablation [27]. By replacing a portion of DPDS with BPM to react with HPC, e.g., replacing a portion of dynamic covalent bonds with traditional covalent bonds, the resin HPBDS was synthesized. The CF/HPBDS composite displayed a much smaller negative ablation rate (Table S14). The CF/HPBDS‐1 sample with the lowest mass ablation rate can also withstand long‐time ablation of 40 s and 60 s with the oxy‐acetylene flame (Figure S32 and Table S15). After long‐time ablation, the surface of the samples is still complete without peeling off or cracks, further reflecting the potential of sulfur‐containing phenolic resin as ablation resistance materials.

## 3 Conclusions

By constructing a phenolic resin‐analogous network containing disulfide bonds, a strategy was developed to improve the char yield and ablation resistance of ablation materials by utilizing dynamic covalent bonds. Based on cross‐linking networks rich in aromatic units, disulfide bonds played a unique role throughout the lifetime of this novel PR. The reversible disulfide bonds, on the one hand, improved chain segment mobility, ensuring effective viscosity regulation and formation of an integrated curing network. On the other hand, the network reconstruction ability enabled high‐efficiency healing of the resin matrix and fiber‐reinforced composites. During thermal pyrolysis, cleavage of disulfide bonds produced thiyl radicals to facilitate char formation through retaining carbon‐rich species. Consequently, a renewable PR, CHPDS‐1.7, with a char yield as high as 64.7% was obtained and can fit for versatile processing technology. The corresponding CF/HPDS composites exhibited an excellent ILSS value of 53.2 MPa and mass ablation rate of 0.029 g·s^−1^, respectively. The damaged CF/HPDS composites can be repaired with healing efficiencies exceeding 70%, and clean CF can be recovered from the discarded composite via solvent treatment. The present study resolves the contradiction between reversibility of dynamic covalent bonds and thermal stability of phenolic resin, providing new solutions for developing high‐performance ablation‐resistant composites for aerospace application.

## 4 Experimental

### 4.1 Materials

4,4'‐Disulfanediyldiphenol (DPDS, AR grade, 98%) was purchased from Tianjin Heowns Opde Technologies Co., Ltd. 2,6‐Bis(hydroxymethyl)‐p‐cresol (HPC, AR grade, 97%) was obtained from Shanghai Macklin Biochemical Technology Co., Ltd. 4,4'‐Dihydroxydiphenylmethane (BPM, GC grade, 99%) was purchased from J&K Scientific Ltd. 2,2‐Bis[4‐hydroxy‐3,5‐bis(hydroxymethyl)phenyl]propane (BHP, AR grade, 97%) was obtained from Shanghai Aichun Biotechnology Co., Ltd. Other chemicals, including ethanol, tetrahydrofuran (THF), toluene,N,N‐dimethylformamide (DMF), and hexamethylenetetramine (HMTA) of analytical grade, were all purchased from Tianjin Fuyu Fine Chemical Co., Ltd. THF and DMF were treated with 4 Å molecular sieves before use. The other reagents were used as received.

### 4.2 Synthesis of Resole‐Type PRs

HPDS‐*x* with different DPDS contents was prepared through a one‐pot reaction without solvent, where *x* denotes the molar ratio of HPC to DPDS. In a typical synthesis, HPC and DPDS were mixed at a certain ratio in a 50 mL round‐bottom flask, and the mixture was heated at 115°C with constant stirring to ensure complete mixing. Subsequently, the temperature was slowly decreased to 105°C and maintained for 30 min, and a homogeneous viscous product was obtained (denoted as HPDS‐*x*). Similarly, parallel syntheses were performed as follows: 1) DPDS was replaced with 4,4'‐dihydroxydiphenylmethane (BPM) to react with HPC, and a PR without disulfide bonds was synthesized (HPBM‐*x*, where *x* denotes the HPC to BPM molar ratio); 2) HPC was replaced with a hydrocarbon‐enriched analog, BHP, to react with DPDS, and the product was denoted as BHDS‐*x* (where *x* denotes the BHP/DPDS molar ratio). Unless otherwise specified, HPDS‐*x*, HPBM‐*x*, and BHDS‐*x* all represent the products obtained after 0.5 h of reaction. The detailed synthetic procedures are provided in the Supporting Information (Tables S1 and S2).

### 4.3 Curing and Processing of PRs

The synthesized HPDS*‐x*, HPBM*‐x*, and BHDS*‐x* were cured in a convection oven at 165°C for 2 h under an N_2_ atmosphere, and the cross‐linked samples were denoted as CHPDS‐*x*, CHPBM‐*x*, and CBHDS‐*x*, respectively. CHPC was obtained by direct curing of HPC at 165°C for 2 h. The curing condition (165°C, 2 h) was chosen based on the most commonly used molding condition for traditional phenolic resin, and to enable a parallel comparison of the properties of the cured resin. The CHPDS‐1.7 sheet specimen was reprocessed via grinding and hot pressing; e.g., approximately 3.8 g of the ground CHPDS‐1.7 powder was evenly spread in the bottom of a square mold (the cavity size was 50 mm × 50 mm), and hot pressed at 210°C/8 MPa for 2 h in a home‐made vulcanizing machine. After cooling to room temperature, the mold was removed to obtain the recycled CHPDS‐1.7 block.

### 4.4 Preparation of PR‐Based composites

First, HPDS*‐x* was dissolved in THF to obtain a 50 wt% stock solution, and the HPDS*‐x* solution was coated on CF through dipping, followed by room temperature storage for 12 h and drying at 50°C over 2 h to prepare prepreg. The mass ratio of CF to HPDS*‐x* was maintained as 60:40. Second, the CF prepreg was segmented into pieces of 80 mm × 80 mm and stacked up to 25 layers, followed by hot pressing at 165°C for 2 h using a home‐made vulcanizing machine supported by a hydraulic jack at 8 MPa. After cooling to room temperature, a CF/HPDS*‐x* composite laminate of *ca*. 80 mm × 80 mm × 3 mm was obtained. Similarly, chopped glass fiber (CGF) reinforced HPDS*‐x* composites, CGF/HPDS*‐x*, were prepared by mixing CGFs with 50% HPDS*‐x* solution at the ratio of 3:4, followed by drying to remove the solvent and pressing in a rectangular mold (25 mm × 80 mm) following the aforementioned procedure.

### 4.5 Characterization

The structure of the samples was evaluated using Fourier Transform Infrared Spectroscopy (FTIR) (Bruker TENSOR 27 FTIR spectrometer) in transmission mode using potassium bromide pellets. The scanning range was 4000–400 cm^−1^, the spectral resolution was 4 cm^−1^, and the scanning number was 60. ^1^H nuclear magnetic resonance (NMR) was performed on an NMR spectrometer (600 MHz, Bruker AV) using acetone‐d_6_ as the solvent to confirm the reaction between DPDS and HPC. The composition and molecular weight of the resin were determined via gel permeation chromatography (GPC, Agilent 1260 Infinity II) in series with three columns (PLgel 500 Å, 100 Å, and 50 Å) with a column temperature of 40°C (THF, 0.6 mL·min^−1^).

The gelation time is measured using a gelation tester (RAY‐NJ‐150). Take 1 mL of resin liquid and place it into a flat‐bottomed hole on a constant‐temperature steel plate. Stir until the resin draws threads and breaks, and record the time. Each sample is tested three times in parallel, and the results are averaged. The curing behavior and glass transition of the PRs were evaluated with a differential scanning calorimeter (DSC, Mettler‐Toledo DSC1) with 5–10 mg sample at the heating rate of 10°C·min^−1^ (0–300°C, nitrogen flow of 50 mL·min^−1^). Thermogravimetric analysis (TGA) was performed on a Mettler‐Toledo TGA2 thermogravimetric analyzer to study the thermal stability of the cured PRs. Approximately 5–10 mg of sample was tested at a heating rate of 10°C·min^−1^ from 30°C to 800°C under a nitrogen atmosphere (50 mL·min^−1^). The dynamic modulus and relaxation of the cured HPDS*‐x* were measured using a dynamic mechanical analyzer (DMA, TA‐Q800) with rectangular samples (30 mm × 5 mm × 1.5 mm) in tensile mode. For modulus measurements, the temperature ranged from 30°C to 250°C at a heating rate of 3°C min^−1^. For stress relaxation measurements, a constant strain of 0.5% was set, and the measurement at each temperature started after equilibration for three minutes. In addition, elemental analyses and Raman spectral analysis were used to study the pyrolysis mechanism of the cured PRs, and the details are provided in the Supporting Information. The detailed information for mechanical properties and ablation experiments was also given in the Supporting Information.

## Author Contributions

**Xiaolong Xing**: formal analysis, investigation. **Yu Li**: conceptualization, writing – review and editing, funding acquisition, investigation. **Ling Yue**: software, methodology. **Huan Yang**: investigation, formal analysis. **Chengshuang Zhang**: project administration, resources. **Cheng Bian**: software. **Boyuan Hu**: investigation, writing – original draft. **Ziqi Zhang**: software. **Ruixue Bai**: writing – review and editing. **Xinli Jing**: conceptualization, supervision, project administration, resources.

## Conflicts of Interest

The authors declare no conflict of interest.

## Supporting information




**Supporting File 1**: advs76868‐sup‐0001‐SuppMat.docx.

**Supporting File 2**: advs76868‐sup‐0002‐SuppVideos.zip.

## Data Availability

The data that support the findings of this study are available on request from the corresponding author. The data are not publicly available due to privacy or ethical restrictions.
