# Supplementary material for: Beyond Traditional Phenolics: Disulfide Bonds for Performance Enhancement of Aerospace Ablation‐Resistant Materials from Processing to Recycling
Source: Adv Sci (Weinh). 2026 Aug 3;13(59):e76868. doi: 10.1002/advs.76868 (PMC13430623; doi:10.1002/advs.76868)
Supplement: Supplementary file 1 [file ADVS-13-e76868-s001.docx]

**Beyond traditional phenolics: Disulfide bonds for performance enhancement of ablation materials from processing to recycling**

Yu Li^1^, Boyuan Hu^1^, Xiaolong Xing^3^, Huan Yang^1^, Ziqi Zhang^1^, Cheng Bian^4^, Ruixue Bai^1^, Ling Yue^1^, Chengshuang Zhang^2^* and Xinli Jing^1^*

1. School of Chemistry, Joint Innovation Center of Thermal Protection Materials, Xi’an Jiaotong University, Xi’an, 710049, China

2. Academy of Aerospace Solid Propulsion Technology, Xi’an, 710025, China

3. Xi’an Aerospace Composites Research Institute, Xi’an, 710025, China

4. Xi’an Modern Chemical Research Institute, Xi’an, 710065, China

*Corresponding author

Xinli Jing

School of Chemistry, Joint Innovation Center of Thermal Protection Materials, Xi’an Jiaotong University, Xi’an, 710049, China

E-mail: [xljing@mail.xjtu.edu.cn](mailto:xljing@mail.xjtu.edu.cn).

Chengshuang Zhang

Academy of Aerospace Solid Propulsion Technology, Xi’an, 710025, China

E-mail: [3425756@qq.com](mailto:3425756@qq.com)

**Supporting information**

[1	Raw materials and synthesis	3](#_Toc231366679)

[1.1	Characterization of raw materials	3](#_Toc231366680)

[1.2	Calculation of the electrostatic potential	3](#_Toc231366681)

[1.3	Synthesis of HPDS and the control sample	4](#_Toc231366682)

[1.3.1	Molecular weight measurement	5](#_Toc231366683)

[1.3.2	Rheology behavior study	6](#_Toc231366684)

[2	Curing of the synthesized resins	7](#_Toc231366685)

[2.1	Curing behaviour	7](#_Toc231366686)

[2.2	Curing degree	9](#_Toc231366687)

[2.3	Characterization of the cured structure	11](#_Toc231366688)

[2.3.1	Swelling test	11](#_Toc231366689)

[2.3.2	Raman analyses	12](#_Toc231366690)

[3	Thermal stability	12](#_Toc231366691)

[3.1	Comparison of CHPDS and CHPBM	12](#_Toc231366692)

[3.2	Analysis of the pyrolysis mechanism	14](#_Toc231366693)

[3.2.1	Calculation of bond energy	14](#_Toc231366694)

[3.2.2	Electron paramagnetic resonance (EPR) spectroscopy	15](#_Toc231366695)

[3.3	Comparison with CBHDS	16](#_Toc231366696)

[3.4	Pyrolysis products analysis	17](#_Toc231366697)

[3.4.1	Gas Chromatography/Mass Spectrometry (Py-GC/MS)	17](#_Toc231366698)

[3.4.2	Elemental analysis	18](#_Toc231366699)

[3.5	Comparison with commercial phenolic resin	18](#_Toc231366700)

[4	Mechanical properties	19](#_Toc231366701)

[5	Composite properties	20](#_Toc231366702)

[5.1	Density measurement	20](#_Toc231366703)

[5.2	Micro-CT characterization	21](#_Toc231366704)

[6	Degradation and recycling	21](#_Toc231366705)

[7	Ablation properties	24](#_Toc231366706)

[7.1	20 s ablation testing	24](#_Toc231366707)

[7.2	Long-duration ablation testing	25](#_Toc231366708)

# 1 Raw materials and synthesis

## 1.1 Characterization of raw materials

The structures of HPC and DPDS raw materials were given as follows.

Figure S1. ^1^H NMR spectra of a) DPDS; b) HPC (Acetone-d_6_).

Figure S2. FTIR spectra of HPC and DPDS.

## 1.2 Calculation of the electrostatic potential

The theoretical calculations were performed using density functional theory (DFT) as implemented in the Gaussian 16 software package. The B3LYP functional combined with the 6-311+G(d,p) basis set was employed, and GD3BJ empirical dispersion correction was included to improve the description of π-π stacking and intermolecular interactions in sulfur-containing aromatic systems. In the DFT calculations, stricter convergence criteria for geometry optimization (opt = Tight) and a finer integration grid (Int = SuperFine) were adopted relative to the default settings to enhance computational accuracy. All optimized geometries were confirmed as local minima on the potential energy surface by vibrational frequency analysis (no imaginary frequencies). The molecular surface electrostatic potentials were calculated using the Gaussian software, Hirshfeld atomic charges were computed using the Multiwfn program, and the electrostatic potential distributions were visualized using the VMD software.

Figure S3. Electrostatic potential and charge.

## 1.3 Synthesis of HPDS and the control sample

Table S1. The feeding ratio for synthesis of HPDS-x.

| Sample | $n_{HPC}$:$n_{DPDS}$ | DPDS(g) | HPC(g) | Sulfur content (wt%) |
| --- | --- | --- | --- | --- |
| HPDS-1.4 | 1.4 | 5 | 4.703 | 12.94 |
| HPDS-1.7 | 1.7 | 5 | 5.711 | 11.72 |
| HPDS-2.0 | 2.0 | 5 | 6.718 | 10.71 |
| HPDS-2.3 | 2.3 | 5 | 7.726 | 9.86 |
| HPDS-2.6 | 2.6 | 5 | 8.734 | 9.14 |

Scheme S1. Schematic illustration of the reaction between BPM and HPC.

Table S2. The feeding ratio for synthesis of HPBM-1.7

| Sample | $n_{HPC}$:$n_{BPM}$ | BPM(g) | HPC(g) | Sulfur content（wt%） |
| --- | --- | --- | --- | --- |
| HPBM-1.7 | 1.7 | 5 | 7.140 | 0 |

### 1.3.1 Molecular weight measurement

The correction curve of gel permeation chromatography (GPC) was obtained by the following method: The calibration curve was obtained using polystyrene with different molecular weights as standard samples. The Lg *M* and retention time were plotted and the correction curve equation obtained by polynomial fitting was as follows:

$\lg M=68.1789-5.43251t+0.15259t^{2}-0.00145t^{3}$	Eq S1

Where *M* and *t* were molecular weight and retention time, respectively.

Figure S4. The GPC spectra of reactants.

Table S3. The relative molecular weight of HPDS-1.7 obtained at different reaction times.

| Sample | Reaction duration(h) | M_n_ (g/mol) | M_w_ (g/mol) |
| --- | --- | --- | --- |
| HPDS-1.7 | 0.5 | 544 | 641 |
| HPDS-1.7-1 h | 1 | 615 | 749 |
| HPDS-1.7-1.5 h | 1.5 | 688 | 827 |

### 1.3.2 Rheology behavior study

The viscosity–time and viscosity–temperature relationship of the synthesized PRs were evaluated on an rheometer (MCR 501, Anton Paar, Austria) using 25 mm disposable parallel plates in two modes: a) rotation with a constant speed of 10 rad·s^-1^ and b) oscillation with a constant frequency of 1 Hz and a constant strain of 0.1%.

Figure S5. a) & b): Demonstration of the processability of HPDS-1.7 based on its viscosity-temperature relationship (heating rate: 2 ℃/min) and viscosity-time relationship.

Figure S6. Demonstration of the processability of HPDS-1.7-1.5 h for hot melting molding technology of HPDS-1.7-1.5 h its viscosity-times relationship (heating rate: 2 ℃/min).

# 2 Curing of the synthesized resins

## 2.1 Curing behaviour

The curing behavior of the above synthesized samples were studied with DSC at different heating rates. The initial temperature ($T_{i}$), peak temperature ($T_{p}$), and terminal temperature ($T_{t}$) of the curing reaction were plot against heating rate. The curing temperatures at steady state were determined by extrapolating the curves to heating rate of zero.

The Kissinger equation and Ozawa equation were used to calculate activation energy of the curing reaction. So, a line can be drawn through the experimental data from thermal analysis and the Kissinger’s corrected kinetic equation. Equation S2 expresses Kissinger’s corrected kinetic equation, and Equation S3 expresses Ozawa’s equation:

$\ln\left( \frac{\text{β}}{\text{T}_{\text{P}}^{\text{2}}} \right)\text{=}\ln\left( \frac{\text{AR}}{\text{ E}} \right)-\frac{E_{a}}{\text{R}}\text{×}\frac{\text{1}}{\text{T}_{\text{P}}}$	Eq S2

$\text{E}_{\text{a}}\text{=}\text{-}\frac{\text{R}}{\text{1.052}}\text{×}\frac{\text{d}\left( \text{lnβ} \right)}{\text{dx}\left( \text{1}/{\text{T}_{\text{P}}} \right)}$	Eq S3

where β represents the heating rate (K•min^-1^). $T_{p}$ is the peak temperature (K). $A$ is the pre-exponential factor (min^-1^) which is related to the collision frequency and the fraction of molecules that collide with the correct orientation. R is the ideal gas constant (8.314 J•mol^-1^•K^-1^). *E_a_* is the activation energy (kJ•mol^-1^). Therefore, using the slope of the line**,** the *E_a_* of the sample can be determined.

Figure S7. a) The DSC curves of HPDS-1.7 at different heating rates; b) Linear regression equations of temperature ( *T_i_*, *T_p_*, and *T_t_* )and Ttversus heating rates of HPDS-1.7; c) Ozawa plot from the experimental data at five heating rates for HPDS-1.7; d) Typical Kissinger plot from experimental data at five heating rates for HPDS-1.7.

Table S4. Curing kinetic parameters of HPDS-1.7.

| *β*(K•min^-1^) | $\text{T}_{\text{i}}\text{ (}$℃) | *T_p_* (℃) | $\text{T}_{\text{t}}\text{ (}$℃) | $E_{a}$(kJ∙mol^-1^) |
| --- | --- | --- | --- | --- |
| 2 | 133.3 | 177.1 | 209.7 | 110.2 |
| 5 | 144.2 | 190.2 | 227.5 |  |
| 10 | 152.3 | 200.2 | 233.2 |  |
| 15 | 158.5 | 208.0 | 243.6 |  |
| 20 | 172.2 | 213.0 | 248.3 |  |

Figure S8. a) The DSC curves of HPBM-1.7 at different heating rates; b) Linear regression equations of temperature ($T_{i}$, $T_{p}$ and $T_{t}$) versus heating rates of HPBM-1.7; c) Ozawa plot from the experimental data at five heating rates for HPBM-1.7; d) Typical Kissinger plot from experimental data at five heating rates for HPBM-1.7.

Table S5. Curing kinetic parameters of HPBM-1.7.

| *β*(K•min^-1^) | $\text{T}_{\text{i}}\text{ (}$℃) | *T_p_* (℃) | $\text{T}_{\text{t}}\text{ (}$℃) | $\text{E}_{\text{a}}$(kJ∙mol^-1^) |
| --- | --- | --- | --- | --- |
| 2 | 138.3 | 172.2 | 203.5 | 99.4 |
| 5 | 146.2 | 186.3 | 218.3 |  |
| 10 | 156.0 | 199.0 | 238.8 |  |
| 15 | 160.2 | 205.8 | 243.0 |  |
| 20 | 165.0 | 211.0 | 249.0 |  |

## 2.2 Curing degree

By conducting DSC measurement of the as-synthesized HPDS-1.7 (or HPBM-1.7) and CHPDS-1.7 (or CHPBM-1.7), the degree of curing was calculated based on exothermic enthalpy measurements:

$\alpha=\frac{{\Delta H}_{0}-\Delta H_{R}}{\Delta H_{0}}\times100\%$	Eq S4

Where *α* is the degree of cure, *∆H_0_* is the heat released when the uncured resin is completely cured, and *∆H_R_* is the residual heat of reaction released from the cured HPDS-1.7.

Figure S9. DSC curves of HPDS-1.7 upon heating and the CHPDS-1.7 cured at 165℃ for 2 h.

Figure S10**.** DSC curves of the HPBM-1.7 upon heating, the CHPBM-1.7 cured at 165℃ for 2 h, cured at 165℃ for 2 h followed by 190℃ for 2 h.

Table S6. The curing degree of the CHPDS-1.7 and CHPBM-1.7.

| Sample | CHPDS-1.7  (165℃/2h) | CHPBM-1.7  (165℃/2h) | CHPBM-.7  (165℃/2h+190℃/2h) |
| --- | --- | --- | --- |
| $\alpha$ | 98.7% | 76.4% | 94.6% |

## 2.3 Characterization of the cured structure

### 2.3.1 Swelling test

The swelling ratio and gel fraction of the cured samples were determined by immersing the resin blocks in ethanol, water and toluene for 48 h at room temperature. The original mass of each sample was recorded as *W*_0_*.* After 48 h immersion, the samples were taken out, wiped with filter paper to remove the surface solvent and weighed immediately. The mass of the swollen sample was denoted as *W*_1_. The swollen samples were thoroughly dried under vacuum and weighed (denoted as *W*_2_). The *R_s_* and *F_g_* of the cured samples were calculated using the following equations:

$R_{S}=\frac{W_{1}}{W_{0}}\times100\%$	Eq S5

$F_{g}=\frac{W_{2}}{W_{0}}\times100\%$	Eq S6

Figure S11. Snapshots of CHPDS-1.7 resin blocks before and after immersion in different solvents at room temperature for 48 h.

Figure S12. *F_g_* and *R_s_* of CHPDS-1.7 after 48 h immersion in various solvents at room temperature.

### 2.3.2 Raman analyses

Raman spectra were recorded using a Lab RAM HR Evolution Raman spectrometer (Horiba, Japan) using laser irradiation of 785 nm wavelength, an exposure time of 10 s, and a laser energy of 10 mW.

# 3 Thermal stability

## 3.1 Comparison of CHPDS and CHPBM

Figure S13. Thermogravimetric analysis of CHPDS-x: a) TGA curves obtained in N_2_ atmosphere; b) DTG curves.

Figure S14. TGA curves of CHPDS (Each sample is measured in duplicate)

Table S7. Char yield of CHPDS-x with different sulfur contents.

| Sample | Sulfur content (wt%) | W_800℃_(wt%) |
| --- | --- | --- |
| CHPDS-1.4 | 12.94 | 63.4±0.3 |
| CHPDS-1.7 | 11.72 | 64.6±0.1 |
| CHPDS-2.0 | **10.71** | 65.4±0.8 |
| CHPDS-2.3 | 9.86 | 64.7±0.6 |
| CHPDS-2.6 | 9.14 | 64.1±0.6 |

Figure S 15. TGA curves of CHPBM-1.7 after segmental curing. (Curing procedure: 165 °C for 2 h followed by 195 °C for another 2 h.)

## 3.2 Analysis of the pyrolysis mechanism

### 3.2.1 Calculation of bond energy

All data in this study were calculated with the Gaussian 16 software package and were optimized at the B3LYP level of density functional theory (DFT). The basis set 6-311+G(d,p) was selected for all atoms, supplemented with GD3BJ empirical dispersion correction to accurately describe π-π stacking and intermolecular interactions in sulfur-containing aromatic systems. In the DFT calculations, strict structural optimization parameters Opt=(Calcfc, Tight, Recalc=5) and high-precision integration grid Int=SuperFine were used to ensure that all optimized structures achieve true energy minima. Vibrational frequency analysis was computed to ensure the points that the minimum have no imaginary frequency. The interaction energies were calculated with counterpoise (CP) correction for basis set superposition error (BSSE) to improve the accuracy of fragment interaction energies.

Figure S16. The bond energies (kcal/mol) of the model compounds derived from theoretical calculations.

### 3.2.2 Electron paramagnetic resonance (EPR) spectroscopy

The EPR experiments were performed using an EPR spectrometer (Bruker E500) at 25°C using a microwave frequency of 9.86 GHz, microwave power of 1 mW, and modulation amplitude of 100 kHz. The g-value was calculated according to the equation: *hν* = *gβB*, where h is Planck’s constant, *ν* is the frequency, *β* is the Bohr magneton, and *B* is the magnetic field.

Figure S17. EPR spectrum of samples at 25 °C.

## 3.3 Comparison with CBHDS

The HPC was replaced with BHP to react with DPDS at the same synthetic conditions, and the resulted product (BHDS) was cured at 165 ℃ for 2 h (Noted as CBHDS).

Scheme S2. Schematic illustration of the reaction between BHP and DPDS.

Table S8. The feeding ratio for synthesis of BHDS-x

| Sample | $n_{BHP}$:$n_{DPDS}$ | DPDS(g) | BHP(g) | Sulfur content (wt%) |
| --- | --- | --- | --- | --- |
| BHDS-0.50 | 0.50 | 0.50 | 0.352 | 14.74 |
| BHDS-0.60 | 0.60 | 0.40 | 0.337 | 13.62 |
| BHDS-0.85 | 0.85 | 0.20 | 0.239 | 11.44 |
| BHDS-1.10 | 1.10 | 0.20 | 0.309 | 9.86 |

Figure S18. Thermogravimetric analysis of the cured BHDS-x samples (CBHDS-x): a) TGA curves obtained in N_2_ atmosphere; b) DTG curves.

Figure S19. TGA curves of CBHDS (Each sample is measured in duplicate)

Table S9. Char yield (800 °C) of CBHDSs with different sulfur contents.

| Sample | Sulfur content (wt%) | W_800℃_ (wt%) |
| --- | --- | --- |
| CBHDS-0.50 | 14.74 | 60.8±0.2 |
| CBHDS-0.60 | 13.62 | 63.2±0.2 |
| CBHDS-0.85 | 11.44 | 58.7±0.1 |
| CBHDS-1.10 | 9.86 | 57.2±1.3 |

## 3.4 Pyrolysis products analysis

### 3.4.1 Gas Chromatography/Mass Spectrometry (Py-GC/MS)

The Py-GC/MS analyses were performed using EGA3030D tubular furnace cracker. The sample injection quantity was 0.5 g and the pyrolysis temperatures were 500 ℃ and 700 °C, respectively. The gas was analyzed by GC/MS-QP2010NX, and the volatilizations with m/z values between 25 and 400 au were taken as the Py-GC/MS analysis results.

### 3.4.2 Elemental analysis

The elemental analysis was carried out on an elemental analyzer (Elementar Instruments Co., Ltd., Germany) to determine the contents of C, H, O, and S in the resin. Measurement conditions: The sample mass was 1–2 mg, and the test temperature was 1150 °C.

Table S10. The results of elemental analysis of the samples.

| Elements | CHPDS-1.7 | CHPBM-1.7 | CHPDS-1.7-treated at 800 °C for 2 h | CHPBM-1.7-treated at 800 °C for 2 h |
| --- | --- | --- | --- | --- |
| C(wt%) | 66.09 | 77.28 | 93.14 | 96.15 |
| H(wt%) | 4.9 | 5.79 | 1.05 | 0.99 |
| O(wt%) | 16.07 | 16.93 | 3.41 | 2.86 |
| S(wt%) | 12.94 | — | 2.43 |  |

## 3.5 Comparison with commercial phenolic resin

In order to verify the contribution of disulfide bond to the char yield of phenolic resin, DPDS was introduced into a commonly used barium-catalyzed phenol-formaldehyde resin (noted as PF) and cured at the same condition as for CHPDS. The cured products (noted as CPFDS-x) was taken for TGA measurement.

Figure S20. The TGA curves and DTG curves of the cured PF resin modified with different mass ratio of DPDS

Table S11. The composition of DPDS modified barium-catalyzed phenol-formaldehyde resin

| Sample | $m_{\text{PF}}$:$m_{\mathrm{DPDS}}$ | DPDS feeding content  (wt%) | Sulfur content  (wt%) | Char yield*  (wt%) |
| --- | --- | --- | --- | --- |
| CPF | — | 0 | 0 | 63.7 |
| CPFDS-2.0 | 2.0 | 33.33 | 8.55 | 65.3 |
| CPFDS-1.7 | 1.7 | 37.04 | **9.50** | **65.7** |
| CPFDS-1.4 | 1.4 | 41.67 | 10.66 | 63.5 |
| CPFDS-1.1 | 1.1 | 47.62 | 12.19 | 61.5 |
| CPFDS-0.8 | 0.8 | 55.56 | 14.22 | 58.9 |

* The reproducibility of TGA is good with system error of 0.2% to 1%.

# 4 Mechanical properties

The tensile strength of the CHPDS*-x* specimens, the interlaminar shear strength (ILSS, according to ISO 178:2001 standard), and flexural strength (according to ISO 14130:1997 standard) of the composite specimens were evaluated on an electronic universal testing machine (CMT 6503, Shenzhen Sansi Testing Instrument Co., Ltd.). For tensile property evaluation, the sample was segmented into pieces with a size of 50 × 5 × 1.5 mm^3^, and the tensile rate was 1 mm·min^-1^.

Figure S21. The stress-strain curves of the samples: a) CHPDS-1.7; b) CHPDS-2.0.

Table S12. The mechanical properties of samples.

| Sample | Tensile Modulus (GPa) | Tensile Strength (MPa) | Elongation at Break (%) |
| --- | --- | --- | --- |
| HPDS-1.7 | 2.0±0.5 | 42.3±0.4 | 3.5±0.4 |
| HPDS-2.0 | 1.4±0.5 | 61.7±7.7 | 7.3±1.3 |

The relaxation time (*τ*) is defined as the time required for the modulus to relax to 1/e of its initial value. The relaxation time and the activation energy conform to the Arrhenius equation:

$ln\tau=\frac{E_{a}}{RT}-lnA\times100\%$	Eq S7

Where *τ* represents the relaxation time, *E_a_* is the activation energy, and *R* is the standard gas constant.

# 5 Composite properties

## 5.1 Density measurement

The densities of composites were obtained on a buoyancy method. The test conditions referred to the ASTM D792 standard, and the liquid and temperature used were water and 23 °C.

Figure S22. The self-healing efficiency of CGF/HPDS-1.7.

Figure S23. Interlaminar Shear Strength of CF/HPDS-1.7(Five measurements were done for one sample).

Table S13. The ILSS recover efficiency for three parallel CF/HPDS-1.7 specimen.

| Sample | 1 | 2 | 3 |
| --- | --- | --- | --- |
| *ρ* (g/cm^3^) | 1.73 | 1.58 | 1.61 |
| ILSS recover efficiency (%) | 52.3 | 76.7 | 51.6 |

## 5.2 Micro-CT characterization

Using a 3D X-ray microscope (Zeiss/Xradia 610 Versa) with a scanning energy level of 80 KV and 126 μA to scan internal structures. After the scanning including the entire sample is completed, 2035 CT images perpendicular to the braiding direction are obtained.

# 6 Degradation and recycling

A piece of the resin block (*ca*. 4.8 g) was immersed into 96 g of DMF, and the resin completely collapsed after 48 h immersion at room temperature. The mixture was transferred into a 250 mL round-bottom flask, equipped with a condenser, and placed under nitrogen protection. The mixture was magnetically stirred at 140 °C for 4 h and the small solid particles were almost completely dissolved, giving a clear solution. Then DMF was removed from the degradation solution by vacuum distillation at 95 °C. After that, the concentrated resin solution was poured onto a PTFE membrane, and the solution was dried in a vacuum oven at 75 °C to remove the solvent. After most of the solvent had evaporated, the residue was pulverized using a grinder and further dried in a vacuum oven at 75 °C to constant weight, yielding the degraded CHPDS-1.7 as a yellow solid.

Degradation of the CF/HPDS composite was carried out in a similar procedure, and carbon fiber cloth was recycled after the resin degradation.

Figure S24. FTIR spectra of CHPDS-1.7 and degraded CHPDS-1.7.

The X-ray photoelectron spectroscopy data were analyzed using an ESCALAB Xi+ photoelectron spectrometer (Thermofisher Scientific, USA). The fitting peaks of each element were based on the peak of C1s at 284.5 eV.

Figure S25. High resolution XPS spectra of (a) carbon and (b) sulfur elements in CHPDS-1.7 after degradation.

Figure S26. Analysis of the elemental composition of CHPDS-1.7 after degradation.

Figure S27. The molecular weight distribution curve of CHPDS-1.7 after degradation.

Figure S28. TGA curves of the degraded CHPDS-1.7 and after it was cross-linked with 8 phr HMTA at 165 ^o^C for 2 h.

# 7 Ablation properties

## 7.1 20 s ablation testing

According to the GJB323A/96 standard, the cylindrical-shaped specimens of 30 mm in diameter and 10 mm in length were conducted on an oxy-acetylene ablative tester manufactured by the Xi'an Aerospace Composite Research Institute (Xi'an, China). The pressure and flux of acetylene were 0.095 MPa and 1.116 m^3^/h, respectively, while those of oxygen were 0.4 MPa and 1.512 m^3^/h, respectively. The volume ratio of oxygen to acetylene was 1.35: 1; The nozzle diameter was 2 mm, the distance of the specimen surface from the nozzle was 10 ± 0.2 mm, and the heat flux density was 4186.8 ± 418.68 kW·m^−2^; The temperature of the oxyacetylene flame was measured using an optical pyrometer and reached as high as 2800 °C. The samples were placed vertically to the flame for 20 s. The mass and linear ablative rates were calculated by six point average thickness and weight change before and after the tests.

Figure S29. SEM images of carbon fiber reinforced composites after ablation treatment.

Table S14. The ablation resistance of carbon fiber reinforced composites.

| Sample | $n_{HPC}:n_{DPDS}$ | $n_{DPDS}:n_{BPM}$ | Density (g/cm^3^) | Mass ablation rate (g/s) | Linear ablation rate(mm/s) |
| --- | --- | --- | --- | --- | --- |
| CF/HPDS-1.7 | 1.7 | / | 1.54±0.03 | 0.029±0.002 | -0.025±0.007 |
| CF/HPDS-2.3 | 2.3 | / | 1.49±0.03 | 0.039±0.003 | -0.035±0.011 |
| CF/HPBDS-1 | 1.7 | 9 | 1.53±0.02 | 0.026±0.002 | -0.014±0.010 |
| CF/HPBDS-2 | 1.7 | 4 | 1.50±0.01 | 0.030±0.003 | -0.006±0.016 |

Considering the CF/HPDS composite displayed negative linear ablation rate, a part of DPDS was replaced with BPM to synthesis HPBDS resin, and the corresponding composites, *i.e.*, CF/HPBDS-1 and CF/HPBDS-1 were prepared for ablation testing.

## 7.2 Long-duration ablation testing

The CF/HPDS-1.7 was fixed onto a home-made ablation tester and exposure to butane-oxygen flame for 180 s. The back-side temperature and the appearance of the sample before /after testing was recorded.

Figure S30. a) CF/HPDS-1.7 sample image; b) Schematic diagram of CF/HPDS-1.7 ablation; c) CF/HPDS-1.7 sample image after ablation.

Figure S31. Variation of back-side temperature with time for traditional phenolic resin and HPDS-1.7 carbon fiber composites. (Butane flame ablation at 1800 °C)

The CF/HPBDS-1 sample was also taken for ablation with exposure to oxy-acetylene flame for 40 s and 60 s at the same other conditions listed in section 5.3.1. The ablation rate was calculated as follows.

Figure S 32. The appearance and surface morphology of CF/HPBDS-1 after 40 s and 60 s ablation

Table S15. Long-duration ablation performance of CF/HPBDS-1

| Ablation time(s) | Mass ablation rate (g/s) | Linear ablation rate(mm/s) |
| --- | --- | --- |
| 40 | 0.030±0.006 | -0.066±0.028 |
| 60 | 0.024±0.001 | -0.087±0.006 |
